# Reference values for bone mass in young athletes: a cross-sectional study in São Paulo, Brazil

**DOI:** 10.1038/s41598-023-27582-8

**Published:** 2023-01-06

**Authors:** Ulysses Fagundes, Rodrigo Luiz Vancini, Alexandre Aparecido de Almeida, Pantelis Theo Nikolaidis, Katja Weiss, Beat Knechtle, Marilia Santos Andrade, Claudio Andre Barbosa de Lira

**Affiliations:** 1 Graduate Program in Translational Medicine, Federal University of Sao Paulo, São Paulo, Brazil; 2 Center of Physical Education and Sports, Federal University of Espírito Santo, São Paulo, Brazil; 3 Tocantins Federal Institute of Education, Science and Technology, Araguatins, Brazil; 4 School of Health and Caring Sciences, University of West Attica, Athens, Greece; 5 Institute of Primary Care, University of Zurich, Zurich, Switzerland; 6 Medbase St. Gallen Am Vadianplatz, St. Gallen, Switzerland; 7 Department of Physiology, Federal University of Sao Paulo, São Paulo, Brazil; 8 Human and Exercise Physiology Division, Faculty of Physical Education and Dance, Federal University of Goiás, Goiânia, Brazil

**Keywords:** Bone, Metabolism, Paediatric research

## Abstract

Reference values for bone mass in young athletes need to be used for screening purposes, and country/regional reference values should be used to improve precision of comparisons. The aim of the present study was to develop reference values for bone health variables in Brazilian young athletes. The bone mass content (BMC), bone mass density (BMD), and total body less head (TBLH) BMD using dual-energy X-ray absorptiometry were measured in 702 young athletes (327 men and 375 women) aged 8.5–18.5 years, divided into 5 groups, according to their age: group 1 (8.5–10.5 years), group 2 (10.6–12.5 years), group 3 (12.6–14.5 years), group 4 (14.6–16.5 years), and group 5 (16.6–18.5 years). Comparisons between the sexes and ages were performed and age-sex-smoothed reference values were constructed. Male athletes presented high BMC and BMD than female athletes in age groups 3, 4, and 5 (*p* < 0.005) and high TBLH BMD than female athletes in age groups 4 and 5 (*p* < 0.005). Reference values, including the percentiles are presented for the bone health variables of each sex and age group. The age-, sex-, and ethnic-specific reference data for bone variables presented in this study enabled more precise reference data for young Brazilian athletes. These data may assist in monitoring changes during an athletes’ growth and different phases of athletic training.

## Introduction

Osteoporosis is considered a disease of the elderly; however, researchers and clinicians agree that it has a pediatric origin^1,2^. Children and adolescents who do not achieve an optimal peak bone mass at the end of adolescence, when almost 90% of peak bone mass is achieved, are more likely to develop osteoporosis during senescence^3^. Therefore, ensuring maximal bone mineral content acquisition during adolescence is important to attenuate loss of bone mass associated with aging process^4^.

Bone mass acquisition may be impaired by some specific disorders present during childhood, such as cystic fibrosis, type 1 diabetes mellitus, or inflammatory bowel disease^5^. Similarly, a sedentary behavior and percentage of body fat were negatively associated with bone mass density (BMD)^6,7^. Conversely, physical activity during childhood may positively affect bone mass gain. Physical activity in children and adolescents has been demonstrated to result in higher bone mass content (BMC) and BMD than non-active youths^4^. In this context, athletes from different sports also demonstrated higher BMC and BMD than general population^8,9^. Weight-bearing exercises, especially those characterized by high-impact loading, such as multiple high-intensity runs, sprints, turns, and jumps, have a high impact on muscles and bones, which are associated with an increase in absolute and relative bone dimensions^10^. The importance of the impact loading characteristics of physical activity can be evidenced by the higher bone mass that the tennis players present in the dominant upper limb compared to the non-dominant limb^11^ or the higher bone mass that handball players (high-loading impact sports) presented than soccer players (odd-loading impact sports)^4^.

Despite the unquestionable benefits of regular physical activity, there is a condition known as relative energy deficiency in sports (RED-S), frequently observed among highly trained male and female athletes^12,13^. This situation is a consequence of inadequate energy intake relative to exercise energy expenditure associated with sports practice, triggering unfavorable health conditions, compromising bone health, and favoring the risk of bone stress injuries among other possible negative effects, such as gastrointestinal and cardiovascular dysfunction^14,15^.

Therefore, reference values for BMC and BMD in male and female children and adolescent athletes, according to age, need to be used by healthcare providers for screening purposes^16,17^.

Dual-energy X-ray absorptiometry (DXA) is widely considered the preferred method for assessing BMC and BMD in clinical and research situations because of the reliability of the results and security of the method (low radiation)^18,19^. In Brazil, sports DXA utility, especially among young people, is currently limited, owing to a lack of normal reference values for Brazilian children and adolescent athletes. Despite the existence of normative data for other populations^20^, Brazil has particular characteristics due to continental dimensions, which are characterized by an enormous diversity of ethnicities and a highly admixed population^21^, resulting from five centuries of colonization and interbreeding among Native Americans, Europeans, and Africans^22^. Therefore, reference values for this population, which have particular characteristics, need to be established.

Therefore, this study aimed to develop normal reference values for total body BMD, BMC, and total body less head (TBLH) BMD, and to construct percentile curves for Brazilian children and adolescent athletes. In addition, the study also aimed to compare bone measurements between sexes. We hypothesized that the male athletes will present higher bone measurements values than the female after the puberty.

## Methods

### Study design

This cross-sectional study involved young athletes who were trained at the Olympic Training and Research Center (São Paulo, Brazil). Each athlete visited the laboratory once for evaluation of body composition.

### Ethics approval and consent to participate

This study was approved by the Human Research Ethics Committee of the Federal University of São Paulo (Brazil) (approval number: 29607020800005505) and conformed to the principles outlined in the Declaration of Helsinki. As the study involved the analysis of the Physiology Laboratory from the Olympic Training and Research Center database, and the data were anonymous, the requirement for informed consent was waived, according to the Human Research Ethics Committee of the Federal University of São Paulo (Brazil). All data were analyzed as the principle of respect for persons, which encompasses the guarantee of privacy, confidentiality, and anonymity rights.

### Participants

A total of 702 athletes (327 men and 375 women) from the Olympic Training and Research Center in São Paulo, Brazil participated in the study. There are several training centers in São Paulo whose athletes compete in federative championships. Athletes who stand out in smaller training centers are sent to train at the Olympic Training and Research Center due to excellent physical structure that is composed by sportive courts, gym, soccer field, athletics track, swimming poll, exercise physiology laboratory, and medical and nutrition division. Thus, the Olympic Training and Research Center is characterized by being frequented by well physical fitness athletes and by athletes who have a wide range of socioeconomic profiles. These reasons make the Olympic Training and Research Center a very representative place for the São Paulo population of child and adolescent athletes. The participants were divided into 5 groups according to their age: group 1 (8.5–10.5 years), group 2 (10.6–12.5 years), group 3 (12.6–14.5 years), group 4 (14.6–16.5 years), and group 5 (16.6–18.5 years). Data were collected between January and December 2015.

A nutritionist provided individualized guidance and food plans to meet the nutritional needs of all the athletes. The participants were runners (n = 34), soccer players (n = 154), artistic gymnasts (n = 16), handballers (n = 128), judokas (n = 30), wrestlers (n = 85), swimmers (n = 86), and volleyballers (n = 169). All the participants were involved in local and national sports competitions. The athletes were trained five times a week for approximately 1 or 2 h per day through 11 months of the year for at least one year. All athletes performed at least 30 min of preventive exercises twice a week, which are exercises performed to improve muscle mass and flexibility. Evaluation of body composition was performed annually for all athletes trained at the Olympic Training and Research Center in São Paulo. Exclusion criteria were as follows: body mass > 130 kg, younger than 8.5 years or older than 18.5 years, height higher than 2 m, pregnancy, and training for less than a year.

Reference values for non-athletes were obtained from a previously published study on healthy children and adolescents^23^. Age, total body mass, height, and body mass index (BMI) of female and male athletes are presented in Table 1.

**Table 1:** Descriptive values for age, height, body mass, and body mass index (BMI) of female and male athletes by age group.

Age group	Age (years old)	Height (cm)	Body mass (kg)	BMI (kg/m^2^)
**Female groups**				
Group 1 (n = 10)	9.6 ± 0.5	138.0 ± 4.1	35.8 ± 4.9	18.7 ± 2.0
Group 2 (n = 41)	11.6 ± 0.5	152.5 ± 8.9	42.9 ± 11.8	18.2 ± 3.3
Group 3 (n = 85)	13.7 ± 0.6	160.9 ± 6.3	54.3 ± 9.0	20.8 ± 2.7
Group 4 (n = 151)	15.5 ± 0.6	164.6 ± 6.8	60.1 ± 10.8	22.1 ± 3.4
Group 5 (n = 88)	17.3 ± 0.5	164.4 ± 6.3	59.6 ± 7.6	22.0 ± 2.2
**Male groups**				
Group 1 (n = 27)	9.3 ± 0.6	134.2 ± 7.2	30.8 ± 6.3	16.9 ± 2.1
Group 2 (n = 50)	11.6 ± 0.6	153.7 ± 11.0	44.5 ± 10.8	18.6 ± 3.0
Group 3 (n = 104)	13.6 ± 0.6	171.2 ± 10.7	62.4 ± 13.4	21.2 ± 3.4
Group 4 (n = 80)	15.6 ± 0.5	177.6 ± 8.8	68.7 ± 12.5	21.7 ± 3.2
Group 5 (n = 66)	17.3 ± 0.6	180.9 ± 7.7	72.0 ± 9.3	21.9 ± 2.3

Data are presented as mean ± standard deviation. BMI: body mass index.

### Body composition evaluation

A DXA (software version 12.3, Lunar DPX, Wisconsin, USA) was used to measure whole-body BMD (g/cm^2^), BMC (g), and TBLH BMD (g/cm^2^)^24^ As the head represents a large portion of the total body mass that changes minimally during growth, it has been excluded from BMD analysis in childhood^25^. The equipment used in the present study could measure total body BMD with a precision of better than 1%^26^.

All tests were performed with the participants wearing comfortable clothes, without metal pieces, centrally aligned with 10 cm between the feet, and 5 cm between the hands and trunk. The participants assumed a supine position. All the tests were performed by the same examiner.

### Statistical analysis

Values are presented as means and standard deviations. Percentile values (10th, 25th, 50th, 75th, and 90th percentiles) for variables related to bone mass are also presented. Two-way analysis of variance (ANOVA) was used to compare BMD, BMC, and TBLH BMD among the age and sex groups, and it was supplemented by Tukey’s post-hoc test when the threshold of significance was reached. Statistical analysis was performed using SPSS v 21.0 (Chicago, Illinois, USA). In all comparisons, *p* values < 5% were considered statistically significant.

## Results

**Figure 1:**
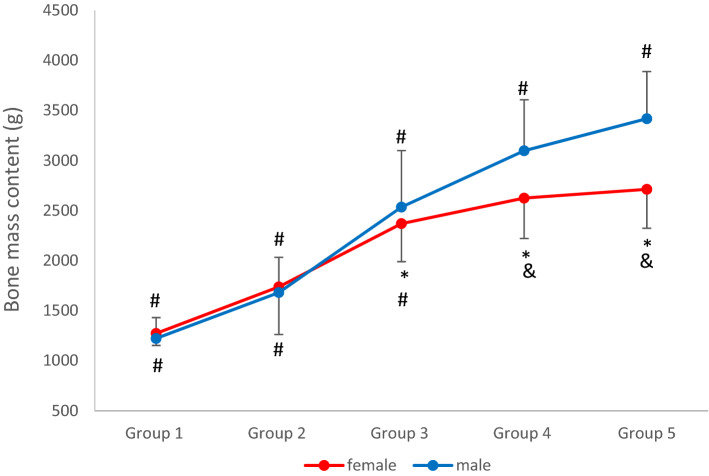
Age- and sex-specific mean values for bone mass content. *p < 0.05 (different from male values for the same age). ^#^*p* < 0.05 (different from all other groups for the same sex). ^&^*p* < 0.05 (different from groups 1, 2, and 3 for the same sex).

The BMC of the male athletes were significantly different among all age groups. In the female group, the BMC for groups 1, 2, and 3 were significantly different from all the other groups, but the BMCs for the age groups 4 and 5 were different only in groups 1, 2, and 3, and were similar between them (Table 2 and Fig. 1). The BMC of the male athletes was significantly higher for the male athletes than that of the female athletes for age groups 3, 4, and 5.

**Table 2:** Age- and sex-specific reference mean and percentiles for body mass content (BMC) (g).

Sex	Age group	BMC (g)	Percentiles				
			10th	25th	50th	75th	90th
Female	Group 1 (n = 10)	1271.9 ± 120.0^#^	1099.6	1194.5	1251.6	1352.7	1492.5
	Group 2 (n = 41)	1736.8 ± 474.7^#^	1206.6	1362.7	1668.6	2026.4	2570.9
	Group 3 (n = 85)	2369.3 ± 380.4*^#^	1939.8	2093.8	2382.9	2591.6	2809.8
	Group 4 (n = 151)	2623.9 ± 403.1*^&^	2114.4	2347.2	2569.5	2848.0	3158.8
	Group 5 (n = 88)	2712.4 ± 389.6*^&^	2186.0	2428.9	2724.1	2971.1	3199.4
Male	Group 1 (n = 27)	1221.8 ± 208.4^#^	985.2	1066.0	1169.9	1431.1	1506.9
	Group 2 (n = 50)	1680.5 ± 352.1^#^	1271.4	1439.8	1599.5	1903.5	2263.2
	Group 3 (n = 104)	2534.7 ± 563.7^#^	1778.6	2111.1	2546.8	2906.9	3255.7
	Group 4 (n = 80)	3097.3 ± 510.2^#^	2463.7	2736.6	3151.0	3493.5	3593.5
	Group 5 (n = 66)	3417.8 ± 471.1^#^	2774.7	3108.1	3414.4	3754.7	3997.8

Data are presented as mean ± standard deviation. BMC: body mass content.

*p < 0.05 (different from male values for the same age).

^#^*p* < 0.05 (different from all other groups for the same sex).

^&^*p* < 0.05 (different from groups 1, 2, and 3 for the same sex).

**Figure 2:**
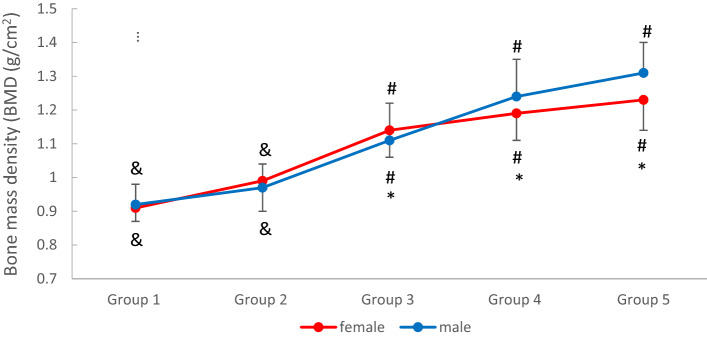
Age- and sex-specific mean values for bone mass density. *p < 0.05 (different from male values for the same age). ^#^*p* < 0.05 (different from all other groups for the same sex). ^&^*p* < 0.05 (different from groups 3, 4, and 5 for the same sex).

The BMD for the male athletes was significantly different among all the age groups. In the female group, the BMDs for groups 3, 4, and 5 were significantly different from all the other groups, but the BMDs for age groups 1 and 2 were different only in groups 3, 4, and 5, and were similar between them (Table 3 and Fig. 2). Conversely, the BMD and TBLH BMD of the male athletes were significantly higher than those of the female athletes only for age groups 4 and 5 (Tables 2, 3, and 4).

**Table 3:** Age- and sex-specific reference mean and percentiles for total bone mineral density (BMD) (g/cm^2^).

Sex	Age group	BMD (g/cm^2^)	Percentiles				
			10th	25th	50th	75th	90th
Female	Group 1 (n = 10)	0.91 ± 0.04^&^	0.86	0.88	0.89	0.95	1.00
	Group 2 (n = 41)	0.99 ± 0.09^&^	0.86	0.91	0.99	1.06	1.14
	Group 3 (n = 85)	1.14 ± 0.08*^#^	1.04	1.09	1.13	1.19	1.26
	Group 4 (n = 151)	1.19 ± 0.08*^#^	1.09	1.13	1.19	1.23	1.29
	Group 5 (n = 88)	1.23 ± 0.09*^#^	1.12	1.16	1.23	1.29	1.34
Male	Group 1 (n = 27)	0.92 ± 0.06^&^	0.83	0.87	0.93	0.94	1.00
	Group 2 (n = 50)	0.97 ± 0.07^&^	0.88	0.92	0.97	1.03	1.07
	Group 3 (n = 104)	1.11 ± 0.11^#^	0.96	1.02	1.12	1.17	1.25
	Group 4 (n = 80)	1.24 ± 0.11^#^	1.08	1.15	1.24	1.32	1.38
	Group 5 (n = 66)	1.31 ± 0.09^#^	1.18	1.23	1.31	1.37	1.42

Data are presented as mean ± standard deviation.

*p < 0.05 (different from male values for the same age).

^#^*p* < 0.05 (different from all other groups for the same sex).

^&^*p* < 0.05 (different from groups 3, 4, and 5 for the same sex).

**Table 4:** Age- and sex-specific reference mean and percentiles for total body less head bone mineral density (TBLH BMD) (g/cm^2^).

Sex	Age group	TBLH—BMD (g/cm^2^)	Percentiles				
			10th	25th	50th	75th	90th
Female	Group 1 (n = 10)	0.81 ± 0.04^&^	0.75	0.78	0.80	0.83	0.89
	Group 2 (n = 41)	0.89 ± 0.09^&^	0.77	0.81	0.89	0.98	1.03
	Group 3 (n = 85)	1.05 ± 0.08^#^	0.96	0.99	1.05	1.10	1.17
	Group 4 (n = 151)	1.09 ± 0.09*^@^	0.98	1.03	1.09	1.13	1.19
	Group 5 (n = 88)	1.12 ± 0.08*^@^	1.00	1.05	1.11	1.18	1.22
Male	Group 1 (n = 27)	0.80 ± 0.09^&^	0.70	0.76	0.79	0.82	0.87
	Group 2 (n = 50)	0.86 ± 0.07^&^	0.76	0.81	0.86	0.92	0.97
	Group 3 (n = 104)	1.04 ± 0.13^#^	0.88	0.95	1.05	1.11	1.18
	Group 4 (n = 80)	1.17 ± 0.11^#^	1.01	1.09	1.18	1.25	1.32
	Group 5 (n = 66)	1.24 ± 0.09^#^	1.11	1.16	1.23	1.29	1.36

Data are presented as the mean ± standard deviation. TBLH BMD: total body less head bone mineral density.

*p < 0.05 (different from male values for the same age).

^#^*p* < 0.05 (different from all other groups for the same sex).

^&^*p* < 0.05 (different from groups 3, 4, and 5 for the same sex).

^@^*p* < 0.05 (different from group 1, 2 and 3 for the same sex).

**Figure 3:**
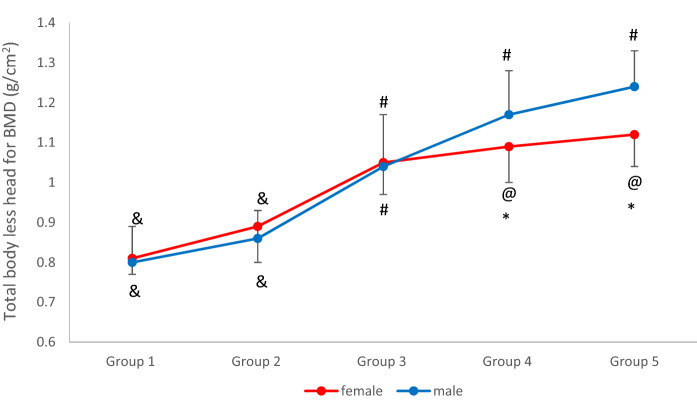
Age- and sex-specific reference mean values for total body less head bone mineral density (TBLH BMD) (g/cm^2^). *p < 0.05 (different from male values for the same age). ^#^*p* < 0.05 (different from all other groups for the same sex). ^&^*p* < 0.05 (different from groups 3, 4, and 5 for the same sex). ^@^*p* < 0.05 (different from group 1, 2 and 3 for the same sex).

The TBLH BMD presented by the male groups 3, 4, and 5 was significantly different from all the other groups, but the age groups 1 and 2 were different only in groups 3, 4, and 5, and were similar between them. Conversely, for the female group and age groups 1 and 2 were different only from groups 3, 4, and 5, and similar between them. Age group 3 was different from all the other age groups, and age groups 4 and 5 were different only from groups 1, 2, and 3, and similar between them (Table 4 and Fig. 3).

**Figure 4:**
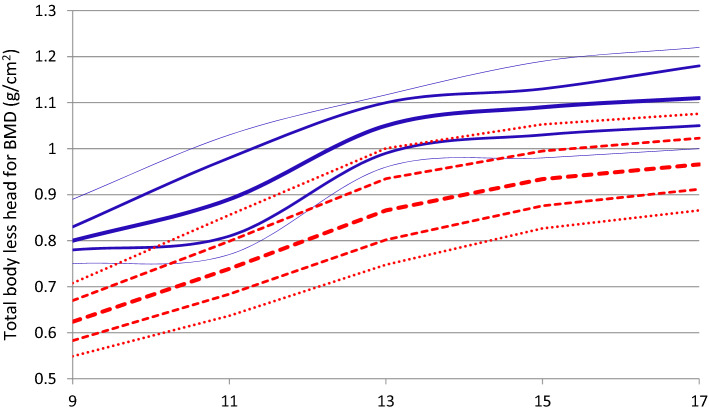
Total body less head BMD in youth female athletes for age groups 1, 2, 3, 4 and 5; percentiles (—) for athletes, and reference percentiles from Lopez-Gonzalez et al.^23^ (---). (the 10th, 25th, 50th, 75th, 90th; and 50th percentiles are in bold).

**Figure 5:**
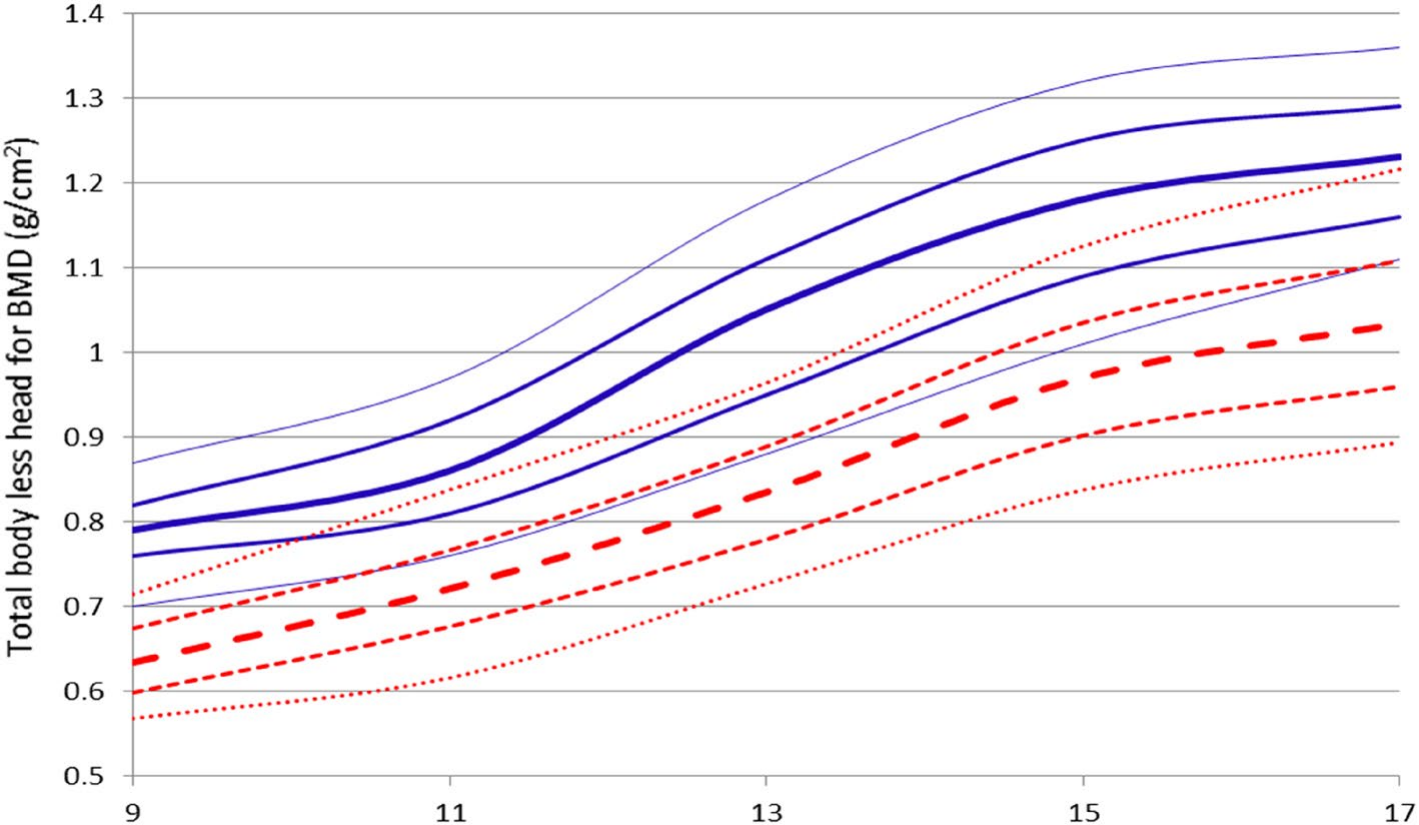
Total body less head BMD in youth male athletes for age groups 1, 2, 3, 4 and 5; percentiles (—) for athletes, and reference percentiles by Lopez-Gonzalez et al.^23^ (---). (the 10th, 25th, 50th, 75th, 90th; and 50th percentiles are in bold).

Female and male data for the age-smoothed percentile graph of TBLH BMD are presented in Fig. 4 and 5, respectively. Differences in the graphical behavior of our results and reference values for non-athletes^23^ can be observed. The median values (50th percentiles) for TBLH BMD in female and male athletes exceeded the 90th percentile of the reference values for non-athletes.

## Discussion

The main aim of the present study was to present sex- and age-specific reference values for BMD, TBLH BMD, and BMC, using a DXA scanner in Brazilian children and adolescent athletes aged 8.5–18.5 years old. Reference values were also plotted with reference values for healthy non-athletic children and adolescents. Moreover, the study aimed to compare sex and age differences, according to bone mass characteristics. The main results were as follows: (i) BMC and TBLH BMD increased during growth in both sexes, but in the female group, the bone measurements tended to stabilize between group 4 (14.6–16.5 years) and group 5 (16.6–18.5 years); (ii) the boys had higher values for BMD and TBLH BMD than the girls after 14.6 years, and higher values for BMC after 12.6 years; and (iii) the median values (50th percentiles) for TBLH BMD presented by female and male athletes exceeded the 90th percentiles of the reference values for non-athletes.

According to the bone measurements comparison among different ages, the present results showed that BMD and TBLH BMD presented a significant increase after group 2 (10.6–12.5 years) for both the male and female athletes; however, for the female group, the TBLH BMD stabilized after group 4 (14.6–16.5 years), while the bone mass for the male athletes still increased until group 5 (16.6–18.5 years). The same pattern of bone mass evolution during childhood and adolescence was shown by Lopez-Gonzalez et al.^23^ in healthy Mexican children and adolescents. Therefore, our results are consistent with those reported in the literature.

When comparing bone mass between the male and female athletes, sex differences became evident after puberty. After 14–15 years of age (group 4), when most of the male athletes are already in puberty^27^, the male athletes presented significantly higher bone measurements than the female athletes, probably due to the influence of sex-related hormones on the bone mass, which will result in the fact that women reach lower peak bone mass than men; this has been associated with higher osteopenia and stress fracture risk in maturity^23,28–30^.

Brazil has a vast territory, and the population is characterized by an enormous diversity of ethnicities, disparities in lifestyle behaviors and socioeconomic status, and conditions that have been known to contribute to differences in BMD among children and adolescents^25,31,32^; therefore, it is necessary to establish specific reference databases for Brazilian children. Moreover, reference data for athletes’ TBLH BMD, BMD, and BMC are limited, particularly among young individuals^14,33^. Therefore, the reference data presented in this study may contribute to the understanding of bone mass in young Brazilian athletes.

The present data showed that BMD and BMC improved throughout childhood. In a visual graphical analysis of TBLH BMD, the 50th percentile curve for the athletes was higher than the 90th percentile for the non-athletes in both the female (Fig. 4) and male participants (Fig. 5). These data are in consensus with previous literature data, which suggest that children and adolescents who play sports should reach a higher BMD and BMC at the end of the second decade of life, when the bone mass reaches its peak^10,14,34–36^.

Therefore, young athletes should have better bone mass conditions to face the losses resulting from aging, thereby reinforcing the concept that osteoporosis, which is very common among the elderly, is a pediatric disease^37^. This osteogenic property attributed to sports practice during childhood appears to be associated with muscle mass gain. The mechanical load over the bone produced by muscular contraction seems to be an osteogenic stimulus for bone formation^38^. In this direction, it has been considered that the weight bearing exercises, especially those presenting high-loading impacts are the most effective to bone mass gain^4,39^. Conversely, athletes involved with aquatic exercises, such as swimming, synchronized swimming or water polo, show similar BMD values compared to controls^39^. In the sample of the present study participated athletes of running, soccer, artistic gymnastics, handball, judo, wrestling, volleyball, and swimming. Except for swimming, all these sports have impact loading, which is desirable for bone mass gain^39^. The swimming is not an impact loading sport, however, swimmers from this study performed terrestrial exercises with the aim of preventing sports injuries, which has been suggested by previous authors and certainly also affects bone mass positively^39^. Therefore, pediatricians, healthcare providers, and parents/guardians must be aware of this information and should encourage the regular practice of sports during childhood and adolescence in school environments or sports clubs.

These reference data present some limitations. Athletes from different sports modalities were combined, and previous reports showed that bone mass gain can be affected by sports characteristics, mainly according to the impact force specificities. For example, athletes from high-impact sports, such as handball, present higher BMD and BMC than athletes from odd-impact sports, such as soccer^4,10^. Maillane et al.^36^ also demonstrated that different sports, such as soccer, karate, and swimming, presented different results regarding BMD gain. Additionally, the participating athletes in the present study were only from São Paulo City and not from all the Brazilian regions. Conversely, it is important to consider São Paulo as a national migratory reception pole. From the beginning of 1970–2000, São Paulo received approximately 300 thousand people from other states a year, which makes the city's population enormously mixed^40^ Moreover, no previous study has demonstrated that bone mass differs among the Brazilian regions. Even so, a broader range of participants might be needed to better represent the Brazilian population. Nevertheless, we believe that these limitations did not limit our conclusions.

## Conclusion

Young Brazilian female athletes presented with significantly lower BMD than males after 14.5 years; moreover, the BMD did not significantly change between 8.5 and 12.5 years for both sexes, but after 12.5 years, the bone density increased significantly in male and female athletes. Finally, young Brazilian athletes presented with the 50th percentile above the 90th percentile for literature reference data for the non-athletes. To the best of our knowledge, this study is the first to present reference bone mass for physically active Brazilian children and adolescents, and to compare these data with literature references for healthy children.

## Data Availability

All data generated or analysed during this study are included in this published article and its supplementary information files.

## Abbreviations

BMC: Bone mass content
BMD: Bone mass density
TBLH: Total body less head
DXA: Dual-energy X-ray absorptiometry
RED-S: Relative energy deficiency in sports
ANOVA: Analysis of variance
